# Web-based program for sexual and reproductive health education of immigrant women: A scoping review protocol

**DOI:** 10.1371/journal.pone.0298551

**Published:** 2024-05-30

**Authors:** Suhyun Kim, Aeri Jang

**Affiliations:** Department of Nursing, Mokpo National University, Muan-gun, Jeollanam-do, South Korea; National Institute of Public Health, MEXICO

## Abstract

Developing web-based education sexual and reproductive health (SRH) programs for immigrant women is crucial. This scoping review aims to provide basic data for developing more advanced programs by examining web-based educational program literature. This review considers web-based SRH education programs for adult immigrant women and focuses on their characteristics, instructional strategies, and outcome evaluations. Data will be extracted following the Minimum Initial Service Package (MISP) and Kirkpatrick level and summarized to show future-oriented results while documenting web-based approach evidence for educating immigrant women on SRH. It is expected to provide information for web-based education programs to meet the MISP and develop various evaluation methods. As such, the findings can be used to determine the direction and level of SRH education.

## Introduction

Global changes in education, politics, administrations, and medical systems because of the COVID-19 pandemic are occurring in nursing care in hospitals and health education in the community [1]. In particular, the World Health Organization recommends addressing human rights, including sexual and reproductive health (SRH) rights, as a key part of the COVID-19 response [2]. Accordingly, in a chaotic situation such as the pandemic, SRH has emerged rapidly as an important topic in health promotion education that the community must give more attention to [3]. In addition, since non-face-to-face health education is still being provided in the community even after the endemic was declared, web-based SRH education needs to be considered in depth [4].

Sexual and reproductive health was put forward at the International Conference on Population and Development in 1994, where reproductive health was defined as a “state of complete physical, mental, and social well-being and not merely the absence of disease or infirmity, in all matters relating to the reproductive system and its functions and processes” [5]. SRH is an integral aspect of overall health, affecting physical, mental, and social well-being. SRH includes health problems related to reproductive organs and sexual health throughout the life of men and women, such as pregnancy, childbirth, puerperal period, family planning, induced abortion, and sexually transmitted diseases such as Acquired Immune Deficiency Syndrome (AIDS) [6].

Immigrant women have poor access to medical services because of cultural differences and language barriers [7]. In particular, when immigrant women in Korea formed an early multicultural society, there were many cases of Japanese women marrying Korean men based on their religious backgrounds, and many Asian women marrying Korean husbands through marriage brokerage companies [8]. These immigrant women had limitations in using health care services or acquiring health information and knowledge due to cultural and language differences, which led them to experience health threats as well as cultural conflicts within their families [7, 8]. In addition, in Korea, depending on where one lives, people cannot receive appropriate prenatal care or there are areas without obstetrics and gynecology hospitals, so web-based SRH education is needed at a national level [8]. In particular, since SRH is not required only for pregnancy and childbirth, an approach that takes this context into consideration is necessary [5]. Until now, Korean society has approached SRH education for immigrant women with a main focus on pregnancy and childbirth [7]. Now, even though the immigrant women who experienced pregnancy and childbirth have become middle-aged women, health care services are not provided for them [9]. Therefore, the transcultural approach is required for their health education [10, 11]. In the current situation where face-to-face group education is limited since of COVID-19, more effective SRH education methods must be considered.

Web-based education has various advantages, such as cost-effectiveness, repeatable studying, and participation even in remote situations, and thus has been widely used for patient and health education [12, 13]. It has been effective in the web-based education program for Chinese immigrant patients with cardiovascular disease [14] and the culturally tailored online cancer support group program for Asian-American patients with breast cancer [15]. However, there may be differences in the educational effect of web-based education, depending on the trainees or its content. Health education programs in the community were reduced or converted into non-contact education programs, and new efforts are continuously being made to access the programs over the Internet to cope with the pandemic [15–17]. Therefore, existing relevant programs must be considered before developing web-based SRH education programs for immigrant women [17].

A scoping review does not describe the results of individual studies in detail. However, it examines the scope of literature in specific areas and helps determine the population, intervention, comparison, and outcome (PICO) components suitable for the review. It is conducted to investigate the potential scope of topics for a systematic literature review [18]. This review explores web-based SRH education programs for immigrant women. Moreover, through the analysis and review of research documents used in this study, similarities and differences will be conceptually mapped. The study will carry out quantitative and qualitative analysis and perform a scoping review on the results found in other studies. A preliminary search of the Medical Literature Analysis and Retrieval System Online (MEDLINE), the Excerpta Medica Database (EMBASE), PubMed, the Cochrane Database of Systematic Reviews (CDSR), and the Cumulative Index to Nursing and Allied Health Literature (CINAHL) was conducted. No current or ongoing systematic or in-progress scoping reviews or systemic reviews on web-based SRH education programs for immigrant women were identified. This scoping review was undertaken to provide primary data for developing web-based SRH education programs for immigrant women that are accessible without temporal and spatial constraints.

This scoping review aims to discover the characteristics of the web-based SRH education programs developed for immigrant women, the strategies used to promote SRH among immigrant women, and the outcomes evaluated in such programs to confirm effectiveness.

## Materials and methods

This study is a scoping review. Its protocol process will be performed according to the preferred reporting items for systematic reviews and meta-analyses protocols (PRISMA-P) [19]. All procedures will be performed according to the preferred reporting items for systematic reviews and meta-analyses extension for scoping review (PRISMA-ScR) [20]. Systematic Review Registrycan be checked through *OSF (*https://doi.org/10.17605/OSF.IO/NZJDT)

### Inclusion criteria

#### Participants

This scoping review is aimed toward immigrant women and covers studies on the education program to improve their SRH. However, tool development, meta-analysis, and review studies were excluded.

#### Concept

This study examined research on the web-based SRH education programs applied to immigrant women and the strategies that promote SRH education effects among them. However, Cochrane-registered and ongoing studies, studies in which only abstracts have been published, descriptive studies, protocols, and interview and workshop materials for program development were excluded from this study.

#### Context

In this review, studies in which web-based SRH education programs were presented for immigrant women in the community were considered. Subjects who migrated within the same country and programs operated as an in-school curriculum were excluded. Eligible research was not limited to geographic limitations.

#### Types of sources

In this scoping review, studies were not limited to design, and experimental and quasi-experimental designs were considered. Qualitative, observational, and descriptive studies related to the research questions were also included.

### Methods

#### Search strategy

This study discussed keyword selection for searches with three search experts and was selected as follows [21]. P of PICO used the mesh terms of “Emigration and immigration,” “Transients and migrants,” “Emigrants and immigrants,” “Refugees,” “Women,” and “Female.” I of PICO used the mesh terms of “Reproductive Health,” “Reproductive,” “Health Services,” “Health Education,” “Sex Education,” “Internet,” “Internet-Based Intervention,” “Smartphone,” and “Mobile Applications.” MESH entry terms related to MESH terms like Emtree, Emtree Synonyms, and Natural Language were considered. For a broader search, we excluded search terms for CO in additional PICO.A limited initial search was performed on MEDLINE to identify articles on the subject. Text and index terms from related articles were used to develop an overall search strategy for MEDLINE (see S1 Appendix). Search strategies will be tailored to each database included, and reference lists of all included articles in other languages will be screened for further research. Databases to search include MEDLINE, EMBASE, Cochrane Library and CINAHL. However, unpublished studies or gray literature will be excluded from this scope review. Reschedule and investigate due up to dates from the original scoping review. Study/Source of evidence selection. All identified literature resulting from the search will be encoded to EndNote V20.0 (Clarivate Analytics, PA, USA), and duplicates will be removed. Then, the titles and abstracts will be screened against the inclusion criteria by one reviewer with consultation from the research team as needed. The full texts of the relevant studies will be retrieved and screened to ensure that the population, concept, and context adhere to the inclusion criteria. Finally, a full-text review of all selected articles will be reviewed against the inclusion criteria by one reviewer. One reviewer will examine the full-text articles following the “narrow and well-defined” inclusion criteria. Any ambiguities at this stage will be resolved through discussion with other research team members, and reasons for the exclusion of full-text studies will be reported in the final scoping review report. Furthermore, the final report will fully detail the search results with a Preferred Reporting Items for Systematic Reviews and Meta-Analysis (PRISMA) flow diagram [19].

#### Data extraction

Using a data extraction tool developed by both authors, primary extraction and examination will be performed (see S2 Appendix). The tool will include information identifying the selected literature’s general characteristics, such as publication year, author, title, journal, study design, research purpose, data collection, topic, and country. The Minimum Initial Service Package (MISP), education method, participant’s characteristics, theoretical framework, duration, and place will also be examined to identify SRH promotion strategies. Finally, findings, outcome variables, and Kirkpatrick levels identifying outcome assessment will also be part of the review.

#### Data analysis and synthesis

The MISP is an international standard of care and aims to diminish mortality, morbidity, and disability in populations affected by crises, specifically women and girls. In this study, data extraction will be performed according to six areas: “Coordination and Lead on implementation of the response,” “Prevent sexual violence and respond to the need of survivors,” “Prevent the transmission of and reduce morbidity and mortality because of the human immunodeficiency virus (HIV) and other sexually transmitted infections (STIs),” “Prevent excess maternal and newborn morbidity and mortality,” “Prevent unintended pregnancies,” and “Plan for comprehensive reproductive health services” to identify which MISP standard areas were included in the web-based SRH education programs [22]. The purpose of this study is to explore the effects of a web-based program for SRH in immigrant women, and the outcomes of these studies will be analyzed based on the Kirkpatrick Model developed in 1959 to evaluate the effects of training. This study will extract data according to the model’s four levels (Reaction, Learning, Behavior, Results) to identify the web-based SRH education program’s outcome the Kirkpatrick levels [23]. In addition, a pilot review will be carried out for the five studies included in this scoping review.

#### Data presentation

The results of this study, such as the general characteristics of the selected literature, SRH promotion strategies, and characteristics of SRH outcome evaluation, will be presented in a table. Quantitative data will be analyzed using descriptive statistics, and the summaries will be described. The results will be utilized to understand the current development situation of web-based SRH education programs for immigrant women. Therefore, the review’s results will contribute to identifying the development and research direction of the SRH education program for immigrant women and suggesting strategies for developing more effective programs.

## Discussion

This scoping review is limited by not including a planned quality assessment of the records as recommended by the methodological framework; thus, the recommendations may impede implementation. Moreover, synthesizing results from different information sources can often be difficult.

Pandemics, such as COVID-19, threaten the SRH of immigrant women under vulnerable conditions [24, 25]. In these situations, web-based education that can minimize temporal and spatial constraints is effective for SRH education [26]. Accordingly, it is essential to develop web-based education programs to enhance the SRH of immigrant women.

Thus, this study will analyze strategies and effect evaluation methods for more effective program development by reviewing the existing literature before developing the SRH education program for immigrant women. Moreover, the data extracted using the data extraction tool will be analyzed, and the design, strategy, and outcome evaluation method of optimal web-based SRH education programs linked to program development will be discussed. The study will also discuss its results and whether there is a potential sex and gender implication [27]. Any changes to the protocol’s content during the scoping review will be specified in detail, and the limitations of the review will be described. All these results and changes will be shared by Open Science Framework.

## Conclusion

This scoping review study will explore web-based education programs promoting immigrant women’s SRH. The review will document evidence of a web-based approach used to educate immigrant women on SRH. This study is expected to provide helpful information for web-based education programs to meet the areas of MISP and develop various program evaluation methods. The review will be based on determining the direction and level of SRH education moving forward.

## Supporting information

S1 Appendix Search strategy for MEDLINE (Pubmed). (DOCX)

S2 Appendix Data extraction table. (DOCX)

## References

[1] Clari M, Luciani M, Sciannameo V, Berchialla P, Di Giulio P, Campagna S, et al. The impact of the COVID-19 pandemic on nursing care: A cross-sectional survey-based study. J Pers Med. 2021;11(10): Article 945. doi: 10.3390/jpm11100945 34683086 PMC8538569

[2] World Health Organization. 2020 [cited 20 December 2021]. Addressing human rights as key to the COVID-19 response. Available from: https://www.who.int/publications/i/item/addressing-human-rights-as-key-to-the-covid-19-response?fbclid=IwAR1NKbpOxUCodHvNh4tyRDBAU1WlLGpl7GTDyZFxBZwXMLs_bEpG90HLt0I.

[3] Osanan GC, Vidarte MFE, Ludmir J. Do not forget our pregnant women during the COVID-19 pandemic. Women Health. 2020;60:959–962. doi: 10.1080/03630242.2020.1789264 32880229

[4] Huang KY, Kumar M, Cheng S, et al. Applying technology to promote sexual and reproductive health and prevent gender based violence for adolescents in low and middle-income countries: digital health strategies synthesis from an umbrella review. BMC Health Serv Res. 2022; 22, 1373 doi: 10.1186/s12913-022-08673-0 36401323 PMC9675248

[5] United Nations. 1995 [cited 12 December 2021]. Report of the International Conference on Population Development Cairo, 5–13 September 1994. Available from: https://www.un.org/development/desa/pd/sites/www.un.org.development.desa.pd/files/a_conf.171_13_rev.1.pdf.

[6] United Nations Population Fund. 1994 [cited 4 January 2022]. Programme of Action–Adopted at the International Conference on Population and Development, Cairo, 5–13 September 1994. Available from: https://www.unfpa.org/sites/default/files/event-pdf/PoA_en.pdf.

[7] Kim S, Cho Chung H. Adaptation to motherhood in Central Asian-Korean immigrants to Korea: A grounded theory study. J Korean Acad Nurs. 2019;49(6), 677. doi: 10.4040/jkan.2019.49.6.677 31932563

[8] Park S, Kim S, Cho Chung H. Adaptation Process of Korean Fathers within Multicultural Families in Korea. Int. J. Environ. Res. Public Health. 2021;18;5935. doi: 10.3390/ijerph18115935 34073131 PMC8199263

[9] Song JE, Ahn JA. Effect of intervention programs for improving maternal adaptation in Korea: Systematic review. Korean J Women Health Nurs. 2013;19(3):129–141. doi: 10.4069/kjwhn.2013.19.3.129 37684759

[10] Salami B, Salma J, Hegadoren K. Access and utilization of mental health services for immigrants and refugees: Perspectives of immigrant service providers. Int J Ment Health Nurs. 2019;28(1):152–161. doi: 10.1111/inm.12512 29984880

[11] Soriano-Ayala E, Cala VC. A transcultural health education programme led by immigrant adolescents in Southern Spain. Health Promot Int. 2019;34(5):970–980. doi: 10.1093/heapro/day057 30060149

[12] De Oliveira Lima L, Saragiotto BT, Costa LOP, Nogueira LC, Meziat-Filho N, Reis FJJ. Self-guided web-based pain education for people with musculoskeletal pain: A systematic review and meta-analysis. Phys Ther Rehab J. 2021;101: Article pzab167. doi: 10.1093/ptj/pzab167 34174081

[13] Huang C-C, Kuo H-P, Lin Y-E, Chen S-C. Effects of a web-based health education program on quality of life and symptom distress of initially diagnosed advanced non-small cell lung cancer patients: A randomized controlled trial. J Cancer Educ. 2019;34:41–49. doi: 10.1007/s13187-017-1263-y 28780685

[14] Zhang L, Ding D, Gallagher R. P202 Are Chinese immigrants with CVD ready to use web-based health information: A comparative study in Australia. Eur Heart J. 2020;41(Supplement_1): Article ehz872.073. doi: 10.1093/ehjci/ehz872.073

[15] Shireman TI, Adia AC, Tan Y, Zhu L, Rhee J, Ogunwobi OO, et al. Online versus in-person training of community health workers to enhance hepatitis B virus screening among Korean Americans: Evaluating cost & outcomes. Prev Med Rep. 2020;19: Article 101131. doi: 10.1016/j.pmedr.2020.101131 32518742 PMC7272502

[16] Kim Y, Kim K, Hong N-S, Kang SJ, Kim E, Kim J-Y, et al. The role of health committee for health management of rural residents in the COVID-19 epidemic. J Agric Med Community Health. 2021;46(4):218–229. doi: 10.5393/JAMCH.2021.46.4.218

[17] Wu H, Sun W, Huang X, Yu S, Wang H, Bi X, et al. Online antenatal care during the COVID-19 pandemic: Opportunities and challenges. J Med Internet Res. 2020;22(7): Article e19916. doi: 10.2196/19916 32658860 PMC7407486

[18] Armstrong R, Hall BJ, Doyle J, Waters E. ‘Scoping the scope’ of a Cochrane review. J Public Health (Oxf). 2011;33(1):147–150. doi: 10.1093/pubmed/fdr015 21345890

[19] Moher D, Shamseer L, Clarke M, Ghersi D, Liberati A, Petticrew M, et al. Preferred reporting items for systematic review and meta-analysis protocols (PRISMA-P) 2015 statement. Syst Rev. 2015;4: Article 1. doi: 10.1186/2046-4053-4-1 25554246 PMC4320440

[20] Tricco AC, Lillie E, Zarin W, O’Brien KK, Colquhoun H, Levac D, et al. PRISMA extension for scoping reviews (PRISMA-ScR): Checklist and explanation. Ann Intern Med. 2018;169(7);467–473. doi: 10.7326/M18-0850 30178033

[21] Connected-U. 2012 [cited 2 January 2022]. In: Connected-U [Internet]. Available from: http://connectedu.co.kr/.

[22] Lisam S. Minimum initial service package (MISP) for sexual and reproductive health in disasters. J Evid Based Med. 2014;7(4):245–248. doi: 10.1111/jebm.12130 25586453

[23] Kirkpatrick J, Kirkpatrick WK. 2009 April [cited 2 February 2022]. The Kirkpatrick four levels: A fresh look after 50 years 1959–2009. Available from: https://www.cedma-europe.org/newsletter%20articles/misc/The%20Kirkpatrick%20Four%20Levels%20-%20A%20Fresh%20Look%20After%2050%20Years%20(Apr%2009).pdf.

[24] Ivanova O, Rai M, Kemigisha E. A systematic review of sexual and reproductive health knowledge, experiences and access to services among refugee, migrant and displaced girls and young women in Africa. Int J Environ Res Public Health. 2018;15(8): Article 1583. doi: 10.3390/ijerph15081583 30049940 PMC6121882

[25] Johri M, Agarwal S, Khullar A, Chandra D, Pratap VS, Seth A, et al. The first 100 days: How has COVID-19 affected poor and vulnerable groups in India? Health Promot Int. 2021;36(6):1716–1726. doi: 10.1093/heapro/daab050 34002217 PMC8194803

[26] Scull T, Malik C, Morrison A, Keefe E. Promoting sexual health in high school: A feasibility study of a web-based media literacy education program. J Health Commun. 2021;26(3): 147–160. doi: 10.1080/10810730.2021.1893868 33779520 PMC8169563

[27] Heidari S, Babor TF, De Castro P, Tort S, Curno M. Sex and gender equity in research: Rationale for the SAGER guidelines and recommended use. Res Integr Peer Rev. 2016;1: Article 2. doi: 10.1186/s41073-016-0007-6 29451543 PMC5793986

