# Supplementary material for: Web-based program for sexual and reproductive health education of immigrant women: A scoping review protocol
Source: PLoS One. 2024 May 30;19(5):e0298551. doi: 10.1371/journal.pone.0298551 (PMC11139288; doi:10.1371/journal.pone.0298551)
Supplement: S1 Appendix — (DOCX) [file pone.0298551.s001.docx]

Appendix I. Search strategy for MEDLINE (Pubmed).

| No. | Search Query | Search Results (01/29/2022) |
| --- | --- | --- |
| #1 | ((((((("Emigration and Immigration"[Mesh]) OR ("Emigration and Immigration"[TW] OR "Immigration and Emigration"[TW] OR "Chain Migration"[TW] OR "Chain Migrations"[TW] OR "Migration, Chain"[TW] OR "Migrations, Chain"[TW] OR "Emigration"[TW] OR "Emigrations"[TW] OR "Immigration"[TW] OR "Immigrations"[TW] OR "In-Migration"[TW] OR "In Migration"[TW] OR "In-Migrations"[TW] OR "Turnaround Migration"[TW] OR "Migration, Turnaround"[TW] OR "Migrations, Turnaround"[TW] OR "Turnaround Migrations"[TW] OR "Labor Migration"[TW] OR "Labor Migrations"[TW] OR "Migration, Labor"[TW] OR "Migrations, Labor"[TW] OR "Out-Migration"[TW] OR "Out Migration"[TW] OR "Out-Migrations"[TW] OR "Return Migration"[TW] OR "Migration, Return"[TW] OR "Migrations, Return"[TW] OR "Return Migrations"[TW] OR "Settlement and Resettlement"[TW] OR "Resettlement and Settlement"[TW] OR "Temporary Migration"[TW] OR "Migration, Temporary"[TW] OR "Migrations, Temporary"[TW] OR "Temporary Migrations"[TW] OR "Border Crossing"[TW] OR "Border Crossings"[TW] OR "Crossing, Border"[TW] OR "Crossings, Border"[TW] OR "International Migration"[TW] OR "International Migrations"[TW] OR "Migration, International"[TW] OR "Migrations, Internationall"[TW] OR "migration"[TW] OR "human migration"[TW] OR "migration rate"[TW] OR "rate, migration"[TW] OR "residential mobility"[TW])) OR ("Transients and Migrants"[Mesh])) OR ("Transients and Migrants"[TW] OR "Migrants and Transients"[TW] OR "Transients"[TW] OR "Transient"[TW] OR "Nonmigrants"[TW] OR "Nonmigrant"[TW] OR "Squatters"[TW] OR "Squatter"[TW] OR "Migrant Workers"[TW] OR "Migrant Worker"[TW] OR "Worker, Migrant"[TW] OR "Workers, Migrant"[TW] OR "Migrants"[TW] OR "Migrant"[TW] OR "Nomads"[TW] OR "Nomad"[TW])) OR ("Emigrants and Immigrants"[Mesh])) OR ("Emigrants and Immigrants"[TW] OR "Immigrants and Emigrants"[TW] OR "Immigrants"[TW] OR "Immigrant"[TW] OR "Foreigners"[TW] OR "Foreigner"[TW] OR "Aliens"[TW] OR "Alien"[TW] OR "Emigrants"[TW] OR "Emigrant"[TW] OR "migrant"[TW] OR "migrants"[TW])) OR ("Refugees"[Mesh])) OR ("Refugees"[TW] OR "Refugee"[TW] OR "Political Asylum Seekers"[TW] OR "Asylum Seeker, Political"[TW] OR "Asylum Seekers, Political"[TW] OR "Political Asylum Seeker"[TW] OR "Seekers, Political Asylum"[TW] OR "Political Refugees"[TW] OR "Political Refugee"[TW] OR "Refugee, Political"[TW] OR "Refugees, Political"[TW] OR "Asylum Seekers"[TW] OR "Asylum Seeker"[TW] OR "Seeker, Asylum"[TW] OR "Seekers, Asylum"[TW] OR "Displaced Persons"[TW] OR "Displaced Person"[TW] OR "Person, Displaced"[TW] OR "Persons, Displaced"[TW] OR "Internally Displaced Persons"[TW] OR "Displaced Person, Internally"[TW] OR "Displaced Persons, Internally"[TW] OR "Internally Displaced Person"[TW] OR "asylum seeker"[TW] OR "asylum seekers"[TW] OR "evacuee"[TW] OR "Refugee Migration"[TW]) | 663,874 |
| #2 | ((("Women"[Mesh]) OR ("Women"[TW] OR "Girls"[TW] OR "Girl"[TW] OR "Woman"[TW] OR "Women's Groups"[TW] OR "Women Groups"[TW] OR "Women's Group"[TW])) OR ("Female"[Mesh])) OR ("Female"[TW] OR "Females"[TW]) | 9,327,108 |
| #3 | #1 and #2 | 200,963 |
| #4 | (((((((("Reproductive health education"[TW] OR "Sexual Health Education"[TW] OR "Sexual and Reproductive Health Education"[TW] OR "Reproductive health Training"[TW] OR "Sexual Health Training"[TW] OR "Sexual and Reproductive Health Training"[TW] OR "Reproductive health Program"[TW] OR "Sexual Health Program"[TW] OR "Sexual and Reproductive Health Program"[TW]) OR ("Reproductive Health"[Mesh])) OR ("Reproductive Health"[TW] OR "Health, Reproductive"[TW] OR "Sexual health"[TW])) OR ("Reproductive Health Services"[Mesh])) OR ("Reproductive Health Services"[TW] OR "Health Service, Reproductive"[TW] OR "Health Services, Reproductive"[TW] OR "Reproductive Health Service"[TW] OR "Service, Reproductive Health"[TW] OR "Services, Reproductive Health"[TW])) OR ("Health Education"[Mesh])) OR ("Health Education"[TW] OR "Education, Health"[TW] OR "Community Health Education"[TW] OR "Education, Community Health"[TW] OR "Health Education, Community"[TW] OR "Training"[TW] OR "Program"[TW] OR "Education"[TW] OR "workshop"[TW])) OR ("Sex Education"[Mesh])) OR ("Sex Education"[TW] OR "Education, Sex"[TW] OR "Family Planning Training"[TW] OR "Training, Family Planning"[TW] OR "Family Planning Education"[TW] OR "Education, Family Planning"[TW] OR "Family Planning Instructors"[TW] OR "Family Planning Instructor"[TW] OR "Instructor, Family Planning"[TW] OR "Instructors, Family Planning"[TW] OR "sexual education"[TW]) | 1,720,833 |
| #5 | ((((((("Internet"[Mesh]) OR ("Internet"[TW] OR "Internet"[TW] OR "World Wide Web"[TW] OR "Web, World Wide"[TW] OR "Wide Web, World"[TW] OR "Cyberspace"[TW] OR "Cyber Space"[TW] OR "webinar"[TW] OR "internet connection"[TW] OR "on-line seminar"[TW] OR "online semina"[TW] OR "web seminar"[TW] OR "web-based seminar"[TW] OR "online"[TW] OR "on-line"[TW] OR "Cyber"[TW] OR "Online Education"[TW] OR "Real-Time Online Education"[TW] OR "Untact online education"[TW] OR "Untact education"[TW] OR "Untact"[TW])) OR ("Internet-Based Intervention"[Mesh])) OR ("Internet-Based Intervention"[TW] OR "Internet Based Intervention"[TW] OR "Internet-Based Interventions"[TW] OR "Intervention, Internet-Based"[TW] OR "Interventions, Internet-Based"[TW] OR "Web-based Intervention"[TW] OR "Intervention, Web-based"[TW] OR "Interventions, Web-based"[TW] OR "Web based Intervention"[TW] OR "Web-based Interventions"[TW] OR "Online Intervention"[TW] OR "Intervention, Online"[TW] OR "Interventions, Online"[TW] OR "Online Interventions"[TW] OR "Internet Intervention"[TW] OR "Internet Interventions"[TW] OR "Intervention, Internet"[TW] OR "Interventions, Internet"[TW] OR "web-based intervention"[TW] OR "internet-intervention"[TW] OR "online-based intervention"[TW] OR "online-intervention"[TW] OR "web intervention"[TW] OR "Web-based"[TW])) OR ("Smartphone"[Mesh])) OR ("Smartphone"[TW] OR "Smartphones"[TW] OR "Smart Phones"[TW] OR "Smart Phone"[TW] OR "Phones, Smart"[TW] OR "smart phone"[TW] OR "smartphones"[TW])) OR ("Mobile Applications"[Mesh])) OR ("Mobile Applications"[TW] OR "Application, Mobile"[TW] OR "Applications, Mobile"[TW] OR "Mobile Application"[TW] OR "Mobile Apps"[TW] OR "App, Mobile"[TW] OR "Apps, Mobile"[TW] OR "Mobile App"[TW] OR "Portable Electronic Apps"[TW] OR "App, Portable Electronic"[TW] OR "Apps, Portable Electronic"[TW] OR "Electronic App, Portable"[TW] OR "Electronic Apps, Portable"[TW] OR "Portable Electronic App"[TW] OR "Portable Electronic Applications"[TW] OR "Application, Portable Electronic"[TW] OR "Applications, Portable Electronic"[TW] OR "Electronic Application, Portable"[TW] OR "Electronic Applications, Portable"[TW] OR "Portable Electronic Application"[TW] OR "Portable Software Apps"[TW] OR "App, Portable Software"[TW] OR "Apps, Portable Software"[TW] OR "Portable Software App"[TW] OR "Software App, Portable"[TW] OR "Software Apps, Portable"[TW] OR "Portable Software Applications"[TW] OR "Application, Portable Software"[TW] OR "Applications, Portable Software"[TW] OR "Portable Software Application"[TW] OR "Software Application, Portable"[TW] OR "Software Applications, Portable"[TW] OR "tablet application"[TW] OR "Mobile"[TW] OR "Application"[TW]) | 1,189,759 |
| #6 | #3, #4, and #5 | 589 |
