# Supplementary material for: Web-based program for sexual and reproductive health education of immigrant women: A scoping review protocol
Source: PLoS One. 2024 May 30;19(5):e0298551. doi: 10.1371/journal.pone.0298551 (PMC11139288; doi:10.1371/journal.pone.0298551)
Supplement: S2 Appendix — (DOCX) [file pone.0298551.s002.docx]

Appendix II. Data extraction table.

| **Scoping Review Details** | |
| --- | --- |
| General character | Publication year, Author(s), Title, Journal |
| Minimum Initial Service Package | Coordination and lead the implementation of the response  Prevent sexual violence and respond to the need of survivors  Prevent the transmission of and reduce morbidity and mortality due to HIV and other STIs  Prevent excess maternal and newborn morbidity and mortality  Prevent unintended pregnancies  Plan for comprehensive reproductive health services |
| Study Design |  |
| Research Purpose |  |
| Data collection | Country, Duration, Place |
| Education Model |  |
| Theoretical Framework |  |
| Participants Characteristics |  |
| Outcome variable | Kirkpatrick Level 1: Reaction  Kirkpatrick Level 2: Learning (Cognitive, Affective, Psychomotor)  Kirkpatrick Level 3: Behavior  Kirkpatrick Level 4: Results |
| Findings |  |
